# The Role of Paternal Accommodation of Paediatric OCD Symptoms: Patterns and Implications for Treatment Outcomes

**DOI:** 10.1007/s10802-020-00678-9

**Published:** 2020-07-18

**Authors:** Benedetta Monzani, Pablo Vidal-Ribas, Cynthia Turner, Georgina Krebs, Caroline Stokes, Isobel Heyman, David Mataix-Cols, Argyris Stringaris

**Affiliations:** 1 Institute of Psychiatry, Psychology & Neuroscience, King’s College London, London, UK; 2 National and Specialist OCD and Related Disorders Clinic for Young People, South London and Maudsley NHS Foundation Trust, London, UK; 3 Social and Behavioral Science Branch, National Institute of Child Health and Human Development, National Institutes of Health, Department of Health and Human Services, Bethesda, MD USA; 4 Primary Care Clinical Unit, Faculty of Medicine, The University of Queensland, Brisbane, Australia; 5 Social, Genetic and Development Centre, Institute of Psychiatry, Psychology & Neuroscience, King’s College London, London, UK; 6 Psychological Medicine, Great Ormond Street Hospital for Children NHS Foundation Trust, London, UK; 7 Institute of Child Health, University College London, London, UK; 8 Centre for Psychiatry Research, Department of Clinical Neuroscience, Karolinska Institutet, Stockholm, Sweden; 9 Stockholm Health Care Services, Stockholm County Council, Stockholm, Sweden; 10 Mood Brain and Development Unit, Emotion and Development Branch, National Institute of Mental Health, National Institutes of Health, Department of Health and Human Services, Bethesda, MD USA

**Keywords:** Obsessive-compulsive disorder, Pediatric, Family accommodation, Cognitive behavior therapy

## Abstract

Family accommodation (FA) refers to the participation of family members in obsessive-compulsive disorder (OCD) rituals. Most studies have focused on maternal accommodation; consequently, little is known about fathers’ accommodation of OCD. The current study aims to extend the existing literature by examining maternal versus paternal accommodation of OCD symptoms.

The sample consisted of 209 children with OCD (Mean [M] age = 14.1 years) and their parents (N_Mothers_ = 209, N_Fathers_ = 209) who had completed the Family Accommodation Scale- Parent Report (FAS-PR). Paired t-test and chi-square analyses were used to compare FA of OCD symptoms between mothers and fathers. Linear regression was used to examine correlates of maternal and paternal FA and its impact on treatment outcomes.

Mothers reported significantly higher levels of daily FA than fathers. Correlates of maternal and paternal accommodation included OCD symptom severity, emotional and behavioral difficulties, and parent psychopathology. Both maternal and paternal FA significantly predicted worse treatment outcomes.

Both mothers and fathers accommodate child OCD symptoms with high frequency, and in similar ways. Although mothers accommodate to a greater extent than fathers, both maternal and paternal involvement in rituals are a significant predictor of the child’s treatment response. Results emphasise the need to consider the whole family system, including fathers, in understanding and treating OCD in children.

**Electronic supplementary material:**

The online version of this article (10.1007/s10802-020-00678-9) contains supplementary material, which is available to authorized users.

## Introduction

Fathers’ contribution to a child’s development and coping with mental disorders is a largely neglected area (Panter-Brick et al., 2014). Accommodation of children’s obsessive-compulsive disorder (OCD) symptoms by mothers is suggested to have a negative impact on treatment outcomes (Strauss et al., 2015); yet, little is known about the role of fathers in OCD. In this study, we examine the pattern of paternal accommodation of children’s OCD and how it affects treatment outcomes.

OCD is common in children and young people with prevalence estimates ranging between 1% - 4% in epidemiological studies (Heyman et al., 2001, Valleni-Basile et al., 1996). The disorder is associated with marked functional impairment in the young person as well as significant family burden and distress (Amir et al., 2000, Cooper et al., 1996, Calvocoressi et al., 1995). The term family accommodation (FA) has been formally used in the OCD literature to refer to the involvement and participation of family members in an individual’s OCD rituals (e.g. providing reassurance, providing items, assisting in avoidance, modifying routines).

To date, a number of studies have examined FA in children with OCD (Lebowitz et al., 2016). Most studies have reported high rates of accommodation among families of children with OCD, suggesting that up to 60–96% of relatives assist or modify their behaviour to accommodate their child’s OCD symptoms (Bipeta et al., 2013, Caporino et al., 2012, Flessner et al., 2011, Futh et al., 2012, Garcia et al., 2010, Lebowitz et al., 2014, Merlo et al., 2009, Peris et al., 2008, Shafran et al., 1995, Stewart et al., 2008, Storch et al., 2007). In addition to being common, various child- and parent-level factors have been associated with parental accommodation, including OCD symptom severity, functional impairment, child’s internalizing and externalizing symptoms, and parent psychopathology (i.e. maternal anxiety and depression) (Caporino et al., 2012, Flessner et al., 2011, Lebowitz et al., 2014, Merlo et al., 2009, Peris et al. 2008, Stewart et al., 2008, Storch et al., 2007, Strauss et al., 2015, Wu et al., 2016, Wu et al., 2019). A handful of studies across both the pediatric and adult OCD literature have also observed an association between FA and treatment outcomes (Cherian et al. a., 2014, Ferrao et al., 2006, Garcia et al., 2010, Merlo et al., 2009; Rudy et al., 2014), albeit not consistently (e.g. Torp et al., 2015). Specifically, lower FA scores at pre-treatment and greater reduction in FA at post-treatment were associated with better outcomes as indicated by lower OCD severity scores (Ferrao et al., 2006; Merlo et al., 2009; Garcia et al., 2010; Rudy et al., 2014). According to the theoretical cognitive-behavioural therapy (CBT) framework, FA hinders CBT effectiveness by reinforcing OCD fears and avoidance (Peris et al., 2008; Storch et al., 2007). Nevertheless, studies have differed in the extent to which their treatments targeted FA, which might explain the inconsistent findings regarding FA as predictor of treatment outcomes (e.g. Torp et al., 2015).

Despite the increased interest in understanding factors that promote FA, the extent to which patterns of accommodation vary between family members - and specifically between mothers and fathers of young people with OCD - remains unclear. Evidence suggests that mothers and fathers interact differently with their children, with fathers contributing uniquely to their child’s behavioral and psychological development across various mental health diagnoses (Bögels and Phares, 2008; Lewis and Lamb, 2003; Ramchandani et al., 2005, 2013). Yet, the role or response of fathers to their child’ OCD symptoms and treatment outcomes is a largely neglected area of research. Indeed, the available OCD literature have either only involved one parent (most commonly the mother) or clustered together different kinship of family members within the same study. It is possible that there might be a differential response between family relatives to a child’s OCD symptoms. For instance, in the adult OCD literature, it has been found that spouses/partners endorse significantly higher FA scores than other family members, such as parents, siblings, children, and cousins (Gomes et al., 2014), and that the burden of caring for an individual with OCD tends to fall on one family member (Cooper et al., 1996). Of relevance to the present study (albeit only 3 participants in their sample had a diagnosis of OCD), Thompson-Hollands et al. (2014) examined parental accommodation among mothers (*n* = 68) and fathers (*n* = 51) of children with anxiety disorder. The authors found a medium association between mother and fathers reports of accommodation (r = 0.27, *p* = 0.06) and that the vast majority of mothers (97%) as well as fathers (88%) engaged in accommodating behaviours. Thus far, the only study that has directly examined differences between mothers (*N* = 41) and fathers (*N* = 29) in response to children’s (*N* = 43) OCD symptoms (Futh et al., 2012) failed to find differences between parents in understanding, narrative, coping, and distress associated with family accommodation. However, the generalizability of these findings is significantly limited by the small sample size, the qualitative nature of the study, and the self-reported diagnostic status of the child (Futh et al., 2012). To our knowledge, no study has empirically examined maternal and paternal accommodation of symptoms separately in a pediatric sample with OCD. Therefore, the question as to how FA differs between mothers and fathers, and how these differences impact or interact with the child’ symptoms and treatment remains to be addressed.

The current study sought to extend the existing work by examining maternal and paternal FA of child OCD symptoms. The first aim of the current study was to examine whether the frequency, type of accommodating behaviours, and correlates of FA of OCD symptoms differed between mothers and fathers. Based on the limited research (Futh et al., 2012; Gomes et al., 2014), we expected to find no differences in the frequency and type of accommodation between mothers and fathers. Based on previous research examining correlates of FA (Flessner et al., 2011, Gomes et al., 14, Peris et al., 2008, Stewart et al., 2008), various child- and parent-related variables were chosen as candidate correlates, including the child’s age and gender, OCD symptoms severity, internalising and externalising difficulties, depressive symptoms, general functioning, and parents’ psychopathology. OCD symptom severity and parent psychopathology were hypothesised to be associated with parental accommodation based on previous findings (e.g. Flessner et al., 2011, Peris et al., 2008, Storch et al., 2007, Wu et al., 2016). However, due to the lack of studies, no specific hypotheses were advanced with respect to how correlates of FA would differ between mothers and fathers. The second aim of the study was to examine whether the association between FA and CBT outcomes differed between mother- and father-reported FA. Since family accommodation has been linked to reduced treatment response irrespective of kinship of family members, both maternal and paternal accommodation of OCD symptoms were hypothesized to predict poorer treatment outcomes.

## Methods

### Participants

The sample consisted of 209 youth (aged 7–18) and their parents (N_Mothers_ = 209, N_Fathers_ = 209) who were referred to the National and Specialist OCD, BDD and Related Disorders Clinic at the Maudsley Hospital, London, met ICD-10 diagnostic criteria for OCD, and whose parents had both completed the relevant measures of FA at assessment.

All data used in the current study was collected as part of routine clinical practice. Approval for the study was received from the South London and Maudsley Clinical Audit and Effectiveness Committee as an audit. All parents and participants provided consent for consent (i.e., consent to be contacted for research purposes). The majority of young people in this study (91.5%) lived with both parents. One hundred and twenty-four participants (*n* = 124; 59.3%) received CBT at the clinic and had post-treatment data available; the remaining participants (*n* = 85, 40.6%) were referred elsewhere for treatment or post-treatment data were not available (e.g. currently in treatment). Indeed, some of the young people referred to the service were seen for assessment only with the view of providing recommendations for treatment to treating clinicians in generic child mental health services. Given that the location of the specialist service was in London, only those who lived locally enough or who found travel to the clinic feasible were treated at the clinic (i.e. feasibility of travelling to the clinic for weekly sessions). There were no significant differences between participants who had post-treatment data available and those who did not with respect to age, gender, mother-reported FA, and OCD severity (all *p* > .05). However, those without treatment data available scored higher in father-reported FA (Without data, M = 22.4 SD = 11.7, With data, M = 18.3 SD = 13.11, t(207) = 2.35, *p* = 0.02, Effect size [ES, Cohen’s *d*] = 0.26).

This study employs data of a large clinical cohort of patients from the National and Specialist OCD and Related Disorder from Young People at the Maudsley Hospital. A number of articles using data from the same cohort have been published. However, none of the previous studies has focused on family accommodation of OCD as the primary outcome or have included data on paternal accommodation of OCD symptoms.

### Measures

*Family Accommodation Scale –Parent Report (FAS-PR):* the FAS-PR is a modified version of the semi-structured, clinician-administered FAS (Calvocoressi et al. 1995). It is a parent-report 13-item measure that assesses the degree to which parents accommodate their child’s OCD symptoms. The FAS-PR measures both the behavioral involvement of family members in their child’s OCD (e.g., modification of daily routines, participation in rituals) and the level of family distress and disruption associated with this involvement. Individual items are rated on a 5-point Likert scale ranging from 0 (never) to 4 (daily). The scale has been commonly used by researchers to assess FA in OCD. Despite its widespread use, however, there is still no agreement on how the FAS-PR should be scored. To date, different methods have been employed across adult and youth samples with OCD, and no studies have formally compared these models with each other. We therefore performed a series of confirmatory factor analyses to compare the different models employed in the literature (Fig. S1) and select the one with more support for this study (see Supplemental for a description of models and selection criteria). The results suggested that the model from Flessner et al. (2009) and Bipeta et al. (2013), which is a 12-item bifactor model (item 10, which rates distress as a result of accommodating, was not included) with a general factor and 2 first-order factors, fitted the data best for both mothers and fathers (Table S1). Therefore, two subscales (Avoidance of triggers and Involvement in compulsions) and/or the *Total* FAS-PR score were employed in all analyses. Cronbach’s α for the present sample were 0.91 for mothers and 0.92 for fathers. Internal reliability of the two subscales (Avoidance of Triggers and Involvement in Compulsions) was high for mothers and fathers (Cronbach’s alphas 0.83–0.88).

*Children’s Yale-Brown Obsessive-Compulsive Scale (CY-BOCS):* the CY-BOCS is a clinician-rated semi-structured interview for assessment of pediatric OCD severity, with sound psychometric properties (Scahill et al., 1997; Storch et al., 2004). It includes an OCD symptom checklist followed by 10 items assessing severity, with scores ranging 0 to 40. In the current study, the CY-BOCS demonstrated good internal consistency (Cronbach’s α = 0.85).

*Children’s Global Assessment Scale (CGAS)* (Shaffer et al., 1983)*:* the CGAS is a validated and reliable measure of severity of disturbance and adequacy of social functioning. The scale ranges from 1 to 100, with 1 representing the most impaired child and 100 representing the healthiest. Scores above 70 represent healthy functioning. The CGAS has shown reliability between raters and across time, ranging 0.53–0.87 (Bird et al. 1987; Rey et al., 1995; Shaffer et al., 1983), and was used in the current study as a clinician-rated measure of functional impairment.

*Beck Depression Inventory for Youth (BDI-Y)* (Beck et al., 1961; Beck et al., 2005)*:* The BDI-Y is a widely-used 21-item self-report measure for depressive symptoms, which has good internal consistency and test-criterion validity. Total raw scores range from 0 to 63, with higher scores indicating greater symptom severity. Cronbach’s α for the BDI-Y in the present study was 0.92.

*Depression Anxiety Stress Scale (DASS)* (Lovibond et al., 1995)*:* The DASS is a validated 42-item self-report measure of parental psychopathology with 14 items within each subscale assessing symptoms of Depression, Anxiety and Stress. Scores on each subscale range from 0 to 42, with higher scores indicating higher levels of distress. Parents rated the extent to which they experienced the symptom over the past week on a 4-point severity/frequency scale. Cronbach’s α values were 0.97 for both mothers and fathers.

*Strengths and Difficulties Questionnaire (SDQ)* (Goodman et al., 1997): the SDQ is a 25-item questionnaire incorporating 5 subscales capturing emotional difficulties, conduct problems, hyperactivity/inattention, peer problems, and pro-social behavior; total scores range from 0 to 40. The measure is widely used across a range of clinical settings, and has been shown to have good psychometric properties, including good internal consistency and retest stability (Goodman, 2001). The SDQ was completed by mothers only. Cronbach’s α for all SDQ items was 0.83. Cronbach’s α for SDQ emotion subscale was 0.70. Cronbach’s α for SDQ hyperactivity subscale was 0.79. Cronbach’s α for SDQ conduct problems subscale was 0.64. Cronbach’s α for SDQ peer problems subscale was 0.74. Cronbach’s α for SDQ prosocial subscale was 0.79.

### Procedure

All young people and their parents attended an initial diagnostic assessment of approximately three hours with a specialist multidisciplinary OCD and related disorders team. During this assessment, CY-BOCS interviews were conducted by clinical psychologists with specialist expertise in OCD, or assistant or trainee psychologists under close supervision. All CY-BOCS interviews were discussed with the multidisciplinary team and an ICD-10 diagnosis of OCD was assigned according to information gained from the CY-BOCS interview, a parental account of current difficulties, and the developmental history. All self-report measures (including the FAS-PR) were completed by young people and their parents prior to the clinic intake and at the end of treatment.

### Treatment

A total of 124 (59.3%) young people with OCD received CBT treatment at the clinic (Mean number of CBT sessions = 15.05, SD = 5.5), delivered by trained therapists or trainees under close supervision from experienced therapists. The CBT intervention was protocol-driven (see Nakatani et al., 2011) and consisted of weekly sessions incorporating psycho-education on OCD and anxiety, the development of a hierarchy of compulsions, exposure and response prevention (ERP), and relapse prevention. The extent to which parental accommodation was addressed in treatment varied depending on the developmental level of the young person and the level of FA when developing a hierarchy of compulsions. Outcome measures, including the FAS-PR, were administered pre- and post-treatment. Approximately one third of those receiving CBT (35.9%) were also on selective serotonin reuptake inhibitor (SSRI) medication; in most cases medication had reached a stable dose before CBT commenced. Those receiving medication were more likely to present with more severe OCD symptoms (Not on SSRI, M = 25.9 SD = 5.0, On SSRI, M = 28.9 SD = 5.1, t(201) = −4.14, *p* < 0.001, ES = -0.60) *and* more impaired scores on measures of global functioning (Not on SSRI, M = 47.6 SD = 10.0, On SSRI, M = 41.0 SD = 8.3, t(171) = 4.34, *p* < 0.001, ES = 0.69), family accommodation (mother, Not on SSRI, M = 22.6 SD = 12.5, On SSRI, M = 29.0 SD = 11.7, t(201) = −3.62, *p* < 0.001, ES = -0.53; father, Not on SSRI, M = 17.7 SD = 12.5, On SSRI, M = 24.6 SD = 12.1, t(201) = −3.83, *p* < 0.001, ES = -0.56), depression (Not on SSRI, M = 19.3 SD = 9.9, On SSRI, M = 23.0 SD = 12.1, t(185) = −2.85, *p* = 0.005, ES = -0.43), child’s psychopathology (Not on SSRI, M = 15.9 SD = 6.3, On SSRI, M = 19.5 SD = 6.1, t(142) = −3.24, *p* = 0.002, ES = -0.57), and father’s psychopathology (Not on SSRI, M = 16.4 SD = 16.5, On SSRI, M = 24.3 SD = 21.0, t(109) = −2.12, *p* = 0.037, ES = -0.43).

### Statistical Analyses

To compare the frequency and scoring of individual items of FA between mothers and fathers, paired t-test and chi-square analyses were employed using FAS-PR (total and subscale) scores and individual items, respectively. In addition, we tested for measurement invariance of FAS-PR across mothers and fathers; specifically, we examined configural, metric, scalar and residual variance invariance (see Supplemental for methods description).

To examine correlates of FA separately for mother and fathers, correlation analyses were first employed to select variables for inclusion in regression models, with *p* < 0.05 as the criterion for entry. Based on previous research (Storch et al., 2007, Stewart et al., 2008, Peris et al. 2008, Flessner et al., 2011, Wu et al., 2016), variables of interest included baseline OCD symptom severity (CY-BOCS total score), child emotional and behavioural difficulties (SDQ total score and subscales), depressive symptoms (BDI), parental psychopathology (DASS total score and subscales), overall general functioning (CGAS), as well as gender, age and duration of illness. In a second step, a series of multivarible linear regression analyses were performed in which significantly correlated variables were entered as independent variables and maternal and paternal FAS-PR scores as dependent variables.

Finally, to examine the predictive value of maternal and paternal FA on treatment outcomes, we performed a series of linear and logistic regression analyses with mothers and fathers FAS-PR scores as predictors. As linear and logistic regressions assume there is no multicollinearity between independent variables, maternal and paternal FA were examined separately. Whilst controlling for pre-treatment severity and gender, following the results from Rudy et al. 2014, we tested whether maternal and paternal FA predicted: (a) post-treatment CY-BOCS severity score; and (b) ‘treatment response’, defined as a 35% or more reduction in CY-BOCS scores pre- to post-treatment (Mataix-Cols et al., 2016).

All regression analyses were performed using structural equation modelling (SEM) with Full Information Maximum Likelihood (FIML) in MPlus (Muthén and Muthén, 2012). FIML allows using all the available data to generate parameter estimates without the need to discard cases with some missing data. Methodologists currently regard maximum likelihood estimation as a preferred missing data technique (Schafer and Graham, 2002) over traditional missing data handling methods (e.g., discarding cases, multiple imputation).

Finally, a multi-level mixed model examined whether mother’s and father’s FAS-PR scores changed as a result of treatment, and whether there was any difference between mothers and fathers in the response, in models unadjusted and adjusted for changes in CY-BOCS scores.

## Results

**Fig. 1:**
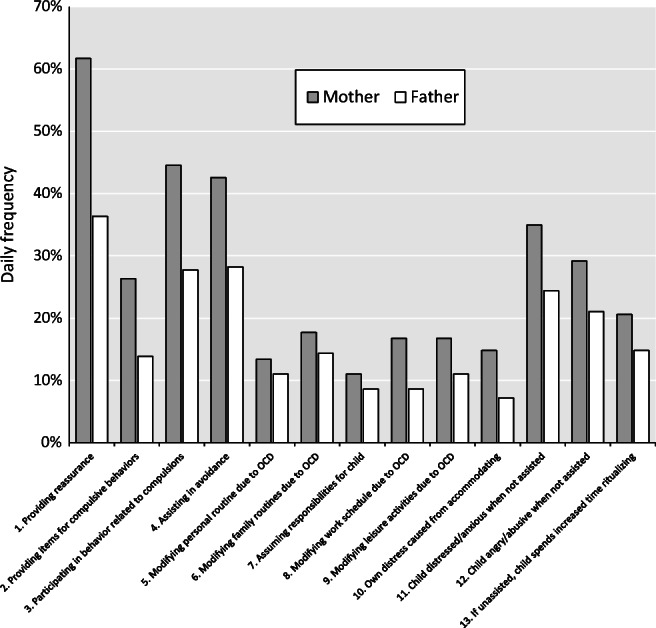
Percentage of mothers (*n* = 209) and fathers (n = 209) endorsing the Family Accommodation Scale-Parent Report (FAS-PR) items daily

Demographic and clinical characteristics of the sample are summarized in Table 1. Parental accommodation was common; 80% of mothers and 57% of fathers reported accommodating their child’s OCD symptoms daily in at least one item. In addition, there was a moderate intra-class correlation (ICC) between mother and father FAS-PR total score (ICC = 0.68, *p* < 0.0001). As shown in Table 2 and Fig. 1, similar patterns of accommodating behaviours were observed for both parents, with provision of reassurance, participation in rituals, and facilitation of avoidance being endorsed most frequently. Relative to fathers, mothers reported significantly higher rates of accommodation on the 12-item FAS-PR Total score (t(208) = 7.59, *p* < 0.0001) and on the subscales of *Avoidance of triggers* (t(208) = 5.14, *p* < 0.0001) and *Involvement in compulsions* (t(208) = 8.32, *p* < 0.0001) .

**Table 1:** Demographic and Clinical Characteristics of the sample (*N* = 209)

**Variable (n)**	**n**	**%**
*Gender (209)*		
*Male*	118	56.46
*Female*	91	43.54
**Variable (n)**	**Mean**	**SD**
*Age, years (209)*	14.10	2.39
*Age of OCD onset, years (192)*	10.42	3.14
*Duration of illness, years (192)*	3.73	2.99
*CY-BOCS Total score (209)*	27.00	5.25
*Obsessions*	13.14	2.82
*Compulsions*	13.86	2.75
*FAS Total score*		
*Mothers (209)*	24.73	12.54
*Fathers (209)*	19.95	12.70
*DASS Total score*		
*Mothers (120)*	26.71	24.26
*Fathers (115)*	18.80	18.53
*SDQ Total score (150)*	17.04	6.43
*BDI (192)*	20.83	10.95
*CGAS (179)*	45.68	10.69

*SD, standard deviation; OCD, Obsessive Compulsive Disorder; CY-BOCS, Children Yale-Brown Obsessive Compulsive Scale; FAS-PR, Family Accommodation Scale Parent Report; DASS, Depression Anxiety Stress Scale; CGAS, Children’s Global Assessment Scale; BDI, Beck Depression Inventory*

**Table 2:** Percentage of mothers and fathers of N = 209 young people with OCD endorsing the Family Accommodation Scale-Parent Report (FAS-PR) items daily

**FAS-PR items**	**Daily frequency**			
	**Mother**	**Father**	**χ**^**2**^	***p*** **value**
1. *Providing reassurance*	*61.7%*	*36.4%*	*31.90*	*<.001*
2. Providing items for compulsive behaviors	26.3%	13.9%	36.90	*<.001*
*3. Participating in behavior related to compulsions*	*44.5%*	*27.8%*	*39.39*	*<.001*
*4. Assisting in avoidance*	*42.6%*	*28.2%*	*27.51*	*<.001*
5. Modifying personal routine due to OCD	13.4%	11.0%	70.27	*<.001*
6. Modifying family routines due to OCD	17.7%	14.4%	57.64	*<.001*
7. Assuming responsibilities for child	11.0%	8.6%	5.66	0.017
8. Modifying work schedule due to OCD	16.7%	8.6%	10.84	0.001
9. Modifying leisure activities due to OCD	16.7%	11.0%	36.09	*<.001*
10. Own distress caused from accommodating	14.8%	7.2%	4.38	0.036
11. Child distressed/anxious when not assisted	34.9%	24.4%	56.17	*<.001*
12. Child angry/abusive when not assisted	29.2%	21.1%	36.36	*<.001*
13. If unassisted, child spends increased time ritualizing	20.6%	14.8%	36.93	*<.001*

*FAS-PR, Family Accommodation Scale Parent Report;* χ^2^_,_ *chi-square; Items in italics denote most frequently endorsed items*

*Note: Item 10, which rates parents’ distress as a result of accommodating, was not included in the analyses based on the results of CFA, and following the model from* *Flessner* et al.*,* 2009*&* *Bipeta* et al.*,* 2013

Configural and metric invariance held, the latter suggesting that the same latent factors were being measured in each rater. However, scalar invariance was not achieved; examination of the modification indices suggested a point of localized strain in the intercept of item 1; accordingly, a partial scalar invariance model was estimated in which the intercept of item 1 (“*How often do you reassure your child?”*) was allowed to differ between groups, resulting in a good-fitting model. This suggests that, at the same level of the latent trait of family accommodation, mothers tend to score higher in item 1 than fathers (Fig. 1, Table 2). A residual variance invariance model held well. Since the threshold for non-invariance in the scalar model was just over the limit (limit: a change of ≤ −0.010 in CFI, supplemented by a change of ≥0.015 in RMSEA *or* a change of ≥0.010 in SRMR; Scalar invariance model comparison: ΔCFI = − 0.011, ΔSRMR = 0.010), and the model itself had an acceptable fit, we conducted the analyses including item 1. However, a sensitivity analysis was conducted excluding this item. Full description and results of measurement invariance can be found in the Supplemental material (Methods, Results, and Table S2).

Based on the number of mothers and fathers who reported daily FA in at least one of the FAS-PR items, there were 114 participants (55%) for whom both parents reported daily FA, 59 participants (28%) for whom only one parent reported daily FA (specifically, 52 mothers and 6 fathers), and 36 participants (17%) for whom neither parent reported daily FA. Bonferroni-corrected one-way ANOVA revealed that those with both parents accommodating daily, compared with those for whom neither parent accommodated daily, scored higher in CY-BOCS (*p* = 0.002), CGAS (*p* < 0.001), BDI (*p* = 0.021), SDQ total score (*p* = 0.003), SDQ emotional (*p* = 0.006), peer problems (*p* = 0.005) and prosocial subscales (reversed) (*p* = 0.008), mother DASS total score (*p* = 0.012), and the depression (*p* = 0.010) and stress subscales (*p* = 0.011), father DASS total score (*p* = 0.002), and anxiety (*p* = 0.006), depression (*p* = 0.014) and stress subscales (*p* = 0.001), and mother (*p* < 0.001) and father FAS-PR (*p* < 0.001). In addition, compared with young people for whom only one parent accommodated daily, those with both parents accommodating daily showed higher scores in SDQ prosocial subscale (reversed) (*p* = 0.003), mother DASS stress subscale (*p* = 0.001), father DASS total score (*p* = 0.047) and stress subscale (*p* = 0.039), and mother (*p* < 0.001) and father FAS-PR (*p* < 0.001).

With regard to correlates of maternal versus paternal FA, no significant correlations were found between parents’ FAS scores and child demographic variables (age, gender, duration of illness) (all *p* > 0.05). In contrast, the child’s OCD symptom severity (CY-BOCS), general functioning (CGAS), depressive symptoms (BDI-Y), child’s emotional and behavioural difficulties (SDQ total score and all subscales expect SDQ hyperactivity) as well as mothers’ and fathers’ psychopathology (DASS total score and their subscales) were significantly correlated with mother and father FA (Table S3). These variables were therefore retained as independent variables and entered into regression models.

The model examining correlates of maternal FAS accounted for 40% of the variance, with OCD symptom severity (β = 0.28, *p* < 0.001), mother DASS (β = 0.34, *p* < 0.001), father DASS (β = 0.15, *p* = 0.045), and SDQ total score (β = 0.16, *p* = 0.028) being significantly associated with maternal FA. With regard to paternal accommodation, the model accounted for 34% of the variance, with OCD symptom severity (β = 0.21, *p* = 0.008), paternal DASS (β = 0.32, *p* < .001), and SDQ total score (β = 0.16, *p* = 0.045) making a significant contribution. BDI and CGAS were not associated with maternal or paternal accommodation of OCD symptoms. Additional exploratory post-hoc analyses were carried out using SDQ and DASS subscales. For maternal FAS, the model accounted for 42% of the variance, however only CY-BOCS total score was significantly associated with FAS (β = 0.26, *p* = 0.001). For paternal FAS, the model accounted for 37% of the variance, with CY-BOCS total score (β = 0.20, *p* = 0.013), SDQ conduct problems (β = 0.21, *p* = 0.012) and CGAS (β = −0.17, *p* = 0.036) being significantly associated with FAS (Table S4).

In terms of treatment outcomes*,* higher maternal FA significantly predicted higher scores on the CY-BOCS at post-treatment (β = 0.27, *p* < 0.001) after adjusting for pre-treatment severity (Table 3). Similarly, father’s accommodation significantly predicted higher post-treatment OCD severity (β = 0.23, *p* = 0.003). Ninety-two participants (74%) met criteria for treatment response. Both mothers’ (OR = 0.96, *p* = 0.037) and fathers’ (OR = 0.95, *p* = 0.004) FA significantly predicted treatment response status, with higher levels of accommodation predicting lower likelihood of response at post-treatment. Similarly, rates of treatment response in participants with two parents accommodating daily (*n* = 63) were lower than in participants with one parent accommodating daily (*n* = 37) or no parent accommodating daily (*n* = 24) (60% vs 84% vs 96%, χ^2^ = 13.98, *p* = 0.001).

**Table 3:** Summary of regression models predicting CBT treatment outcomes

	**Post-treatment OCD severity**				**Treatment Response Status**			
	**β**	**SE**	**z**	***p*** **value**	**OR**	**SE**	**z**	***p*** **value**
Maternal FAS-PR	0.27	0.08	3.6	<0.001	0.96	0.02	−2.08	0.037
Pre-CYOBCS	0.40	0.07	5.5	<0.001	0.90	0.04	−2.52	0.012
Gender	−0.11	0.08	−1.4	0.164	1.34	0.60	0.56	0.577
	R^2^ = 0.32, *p* < 0.001				R^2^ = 0.19, *p* = 0.029			
Paternal FAS-PR	0.23	0.08	3.0	0.003	0.95	0.02	−2.88	0.004
Pre-CYOBCS	0.40	0.08	5.1	<0.001	0.91	0.04	−2.00	0.046
Gender	−0.09	0.08	−1.2	0.236	1.36	0.62	0.57	0.569
	R^2^ = 0.27, *p* < 0.001				R^2^ = 0.21, *p* = 0.013			

*FAS-PR, Family Accommodation Scale Parent Report; CY-BOCS, Children Yale-Brown Obsessive Compulsive; SE, standard error*

Levels of FA decreased after treatment in both mothers and fathers (B = -22.12, *p* < 0.001), with a rater by time interaction (B = 2.79, *p* = 0.039), in which FA decreased slightly more in mothers. In models adjusted for changes in CY-BOCS scores, the decrease in FA was still evident (B = -9.36, *p* < 0.001), however the interaction effect disappeared (B = 1.70, *p* = 0.162). At post-treatment, mothers scored higher than fathers in FA in both unadjusted (B = 1.99, *p* = 0.038) and adjusted models (B = 3.08, *p* = 0.001).

## Discussion

Fathers’ response to their child’ OCD symptoms remains a largely neglected area of research. This is the first and largest quantitative study to date to examine levels and correlates of parental accommodation of OCD symptoms in young people and its association with treatment outcomes with a specific emphasis on investigating and comparing maternal and paternal accommodation.

The results of the current study indicate that *both* parents engage in high and similar patterns of accommodation of their child’s OCD symptoms. Specifically, both mothers and fathers reported provision of reassurance and facilitation of avoidance as the two most frequent types of accommodation provided. Furthermore, for most of our sample (*n* = 114, 55%) both parents accommodated their child’s OCD daily. This subgroup of young people scored higher on measures of OCD severity, functional impairment, child emotional and behavioural difficulties, and parent psychopathology compared to those where only one (*n* = 59, 28%) or no (*n* = 36, 17%) parent accommodated daily. Notably, a moderately high correlation between mothers’ and fathers’ FAS-PR scores was also observed, suggesting that if one parent accommodates a child’s symptoms the other seems more likely to accommodate as well. These findings suggest that parents might take a similar approach to a child’s OCD. Whilst both parents give accounts of being similarly drawn into rituals, parents differed in the levels of symptom accommodation, with mothers accommodating more than fathers. Furthermore, our results indicate that in cases where only one parents accommodates, this tends to be the mother, which might simply relate to the amount of time a caregiver spends with a child with OCD. Although this study did not collect information regarding how much time each parent spent with their child, future studies may benefit from addressing this limitation. Nonetheless, both parents highly accommodate their child’s OCD symptoms, highlighting the importance of considering *both* parents in the assessment and treatment of OCD to ensure that they are both able to withdraw successfully from OCD symptoms rather than having one parent inadvertently maintain a cycle of rituals and avoidance. Our finding that mothers and fathers report a highly similar pattern of accommodation is in line with previous investigations on this topic (Futh et al., 2012; Gomes et al., 2014, Thompson-Hollands et al., 2014). Indeed, these results extend the findings of a recent study on parents of young people with anxiety disorders, whereby the authors found 97% of mothers and 88% of fathers engaged in FA as well as a medium correlation between mother and father reports of accommodation (r = 0.27, *p* = 0.06) (Thompson-Hollands et al., 2014). However, our findings also indicate differences in the extent of FA between parents, with mothers accommodating their child’s OCD symptoms to a far greater extent than fathers. The lack of research comparing mothers versus fathers’ FA and the small number of fathers participating in prior studies in OCD may have provided limited power to detect differences in the extent of FA between parent dyads and supports the scope for further research to address questions concerning paternal role in OCD.

The current study also sought to examine correlates of parental accommodation for mothers and fathers. The finding that OCD symptom severity predicted both maternal and paternal involvement in rituals is not surprising. Indeed, this result emerges consistently as one of the factors most relevant to understanding FA (Storch et al., 2007; Peris et al., 2008) and, furthermore, has been supported by two recent meta-analyses that reported a medium-sized correlation (r = 0.35–0.42) between FA and OCD symptom severity (Strauss et al., 2015, Wu et al., 2016). Moreover, post-hoc analyses in our study indicated that symptom severity remained a significant correlate of both maternal and paternal FA even after taking into account additional measures in the model. Alongside OCD severity, our findings confirmed a relationship between child’s emotional and behavioural difficulties (as measured using the SDQ) and parental psychopathology (as measured by the DASS) with FA. This was found to be equally true for mothers and fathers. In addition, fathers’ distress (DASS) was also associated with mothers FA, though the reverse was not observed. Unfortunately, the causal direction of this association cannot be established. Children with heightened emotional/behavioural difficulties might facilitate parent’s accommodation. Similarly, distressed parents could be more likely to accommodate in response to their child’s difficulties. Yet, the reverse pattern however could also be true; that is, FA could lead to increased child’emotional/behavioural difficulties and/or parental distress. These two alternatives would have different implications for treatment and remain an important question to be addressed in future longitudinal research. The finding that symptom severity exerts an influence on mothers’ as much as on fathers’ likelihood to accommodate strongly supports the need for education for all family members regarding this coercive cycle and management strategies for such behaviour. In addition, our results highlight the potential value of screening and providing additional support for children who present with generalized heightened emotional/behavioural difficulties and/or whose parents present with psychopathology. Our findings also reinforce the need to consider fathers’ perspective as potentially indirectly influencing maternal accommodation.

Clinical practice guidelines currently recommend the involvement of parents according to the needs of the child (NICE, 2005). Certainly, there is a growing body of literature demonstrating good clinical outcomes for family-based CBT, with emerging evidence supporting the additive effect of parental involvement in OCD treatment (e.g. Lebowitz et al., 2020, Storch et al., 2007; Freeman et al., 2014; Rudy et al., 2014). As hypothesized, the results in this study support the association between family accommodation and treatment outcomes. Similar to maternal FA, we found that greater levels of fathers’ accommodation of symptoms predicted more severe OCD symptoms at post-treatment, even after controlling for pre-treatment severity. Moreover, in this study, paternal accommodation was found to be a significant predictor of response status. This is a novel finding that encourages more consideration and research on fathers’ perspective in pediatric OCD. Since fathers are usually less involved in treatment, they may continue to engage in accommodation of OCD symptoms inadvertently maintaining or reinforcing OCD behaviours.

Treatment response was poorer for those participants with both parents accommodating daily (60% response rate) than those with only one (84% response rate) or no (96% response rate) parent accommodating, providing preliminary evidence to support the inclusion of both parents in family-based CBT for OCD. Emerging evidence supports the benefits of family-based CBT, incorporating treatment modules that address FA (e.g. Grunes et al., 2001; Rudy et al., 2014). In a small trial, greater OCD reductions were observed for patients assigned to a behaviour therapy plus family intervention group, compared to those in the behaviour therapy only condition (Grunes et al., 2001). Future research is warranted to examine the additional benefit of involving both parents, as opposed to one relative, in family-based interventions for pediatric OCD. Indeed, whilst common clinical practice tends to involve the participation of one parent in treatment, usually mothers (Iversen et al., 2012), our results provide preliminary support in favor of further consideration of fathers’ role in their child’s OCD treatment. For instance, research has shown that the involvement of fathers in treatment of disruptive behaviors in adolescence can lead to improved treatment outcomes (e.g. Bagner and Eyberg, 2013; Lundahl et al., 2008). In contrast to the literature on externalizing behaviors (Iversen et al., 2012), this issue remains to be examined in anxiety disorders and/or OCD. However, the current findings point to the potential benefit of examining this issue further and investigating the impact of the involvement of fathers in treatment.

A number of shortcomings ought to be considered when interpreting the findings of this study. First, as with previous research examining parental accommodation in paediatric OCD, information on mothers, fathers, and families was limited minimizing our ability to fully understand how parents’ role and involvement might vary based on certain clinical or demographic information (e.g. age, SES, married status, ethnic status). Future research would benefit from collecting further demographic and clinical information on parents. Second, only families where both parents completed the FAS were included in the study. Although the majority of young people in this study (91.5%) were cohabitating with both their mother and father, differential patterns of FA may be observed or be influenced by parents’ living arrangement. Third, whilst data on ethnicity and family composition were not available for all participants, anecdotally the sample consisted of largely White, British families. Therefore, our findings may not be generalizable to a range of families from differing ethnic backgrounds. Fourth, the study relied on parents as central informants and no measures were in place to ensure mothers’ and fathers’ responses on the FAS-PR were collected independently from the other parent. Therefore, these findings should be replicated using observational and clinician-administered measures of FA. Fifth, we did not include a measure of the time that parents spent with their child. It is possible that the difference in the extent of FA between mothers and fathers is simply related to the time spent with their child as opposed to the type of the caregiver, specifically. This requires investigation in future studies. Sixth, only a sub-group of our sample had treatment outcome data available. Although those with and without outcome data were comparable with respect to most variables of interest (with the exclusion of father-reported FA), they may have differed with respect to other clinical characteristics that were not measured in this study. Seventh, the lack of measures of maternal and paternal involvement in the OCD treatment is another limitation in that it does not allow one to assess their respective impact on treatment outcomes. It is possible that the extent to which FA is directly addressed in treatment influences results across studies on FA in OCD; as such, future research examining FA would benefit from careful measuring, monitoring and tracking of parental involvement in treatment. Eighth, post-treatment CY-BOCS was not routinely collected by an independent clinician in all cases. However, it is unlikely that clinicians’ ratings were directly influenced by parent reports of family accommodation. Finally, causality between FA and treatment response cannot be established from this study. More research is needed to confirm the direction of causation in order to inform intervention strategies. This could be addressed in randomized controlled trials which are beginning to emerge (e.g. Grunes et al., 2001; Freeman et al., 2014).

Notwithstanding these limitations, the inclusions of both parents in the examination of FA of OCD is an important contribution to the pediatric OCD literature. The present study found that mothers and fathers are more similar in their propensity to accommodate child OCD than previously thought. The findings also highlight the value of appropriate screening and targeting of both maternal and paternal symptom accommodation in pediatric OCD. Further research is needed to investigate the contribution and involvement of fathers in OCD treatment.

## Electronic supplementary material

ESM 1 (DOCX 132 kb)

## Abbreviations

OCD: Obsessive Compulsive Disorder
FA: Family Accommodation
FAS-PR: Family Accommodation Scale- Parent Report
ICC: intraclass correlations
