# Supplementary material for: The Role of Paternal Accommodation of Paediatric OCD Symptoms: Patterns and Implications for Treatment Outcomes
Source: J Abnorm Child Psychol. 2020 Jul 18;48(10):1313–23. doi: 10.1007/s10802-020-00678-9 (PMC7445192; doi:10.1007/s10802-020-00678-9)
Supplement: Supplementary file 1 — (DOCX 132 kb) [file 10802_2020_678_MOESM1_ESM.docx]

**Supplementary Methods and Results**

**Methods**

*Confirmatory factor analysis (CFA)*

Confirmatory factor analysis (CFA) were employed to evaluate and compare the relative fit of four alternative factor structures or methods for scoring the FAS-PR used across the OCD literature (*Figure S1*). We performed CFA in MPlus version 7 (Muthen and Muthen, 2012), using maximum-likelihood estimation with robust standard errors (MLR). We followed common practice in reporting multiple indices of model fit, namely the Comparative Fit Index (CFI), the Tucker Lewis Index (TLI), and the Root Mean Square Error of Approximation (RMSEA) (Brown, 2006; Hu & Bentler, 1999). To consider a model as showing ‘acceptable’ fit, we required a CFI>0.90, TLI>0.90, and RMSEA<0.08; to consider a model as showing ‘good’ fit, we required a CFI>0.95, TLI>0.95, and RMSEA<0.06 (Brown, 2006). We also compared models using the Akaike Information Criteria (AIC) and the Bayesian Information Criteria (BIC), where lower values indicate better fit (Burnham & Anderson, 2004; Levy & Hancock, 2007). Unlike χ2, AIC and BIC penalize per increasing number of estimated parameters, thus avoiding overfitting models. The best fitting FAS-PR model was then employed for all subsequent analyses.


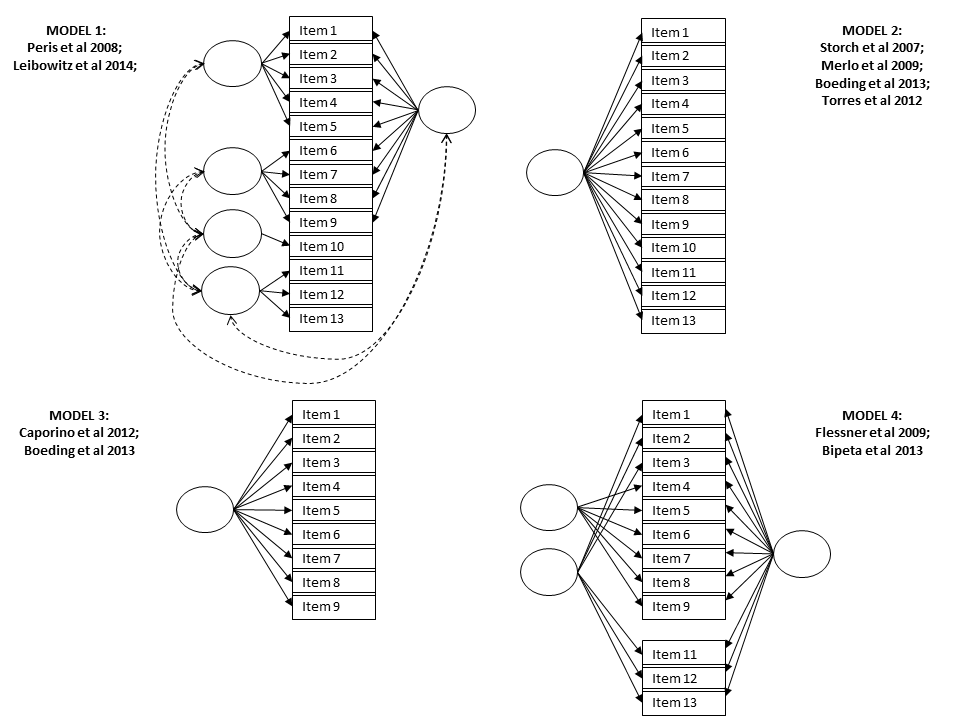


***Figure S1.* *The four factor structures or scoring methods used for the Family Accommodation Scale- Parent Report (FAS-PR) across the OCD literature.*** *Model 1 is a bi-factorial model with a four -first order group factors or subscales (Participation, items 1-5; Modification, items 6-9; Distress, item 10; and Consequences, items 11-13) and a general factor (Total score) loading on Participation and Modification items (Lebowitz et al., 2014; Peris et al., 2008 ). Model 2 (Boeding et al., 2013; Merlo et al., 2013; Peris et al., 2008; Storch et al., 2007; Torres et al., 2012) is a unidimensional model with a single factor including all 13 items. Model 3 is a unidimensional model with a single factor including the first 9 items of the FAS-PR only (Caporino et al 2012; Boeding et al 2013). Model 4 is a bi-factor model with two -first order group factors (Avoidance of triggers, items 4-9; Involvement in compulsions, items 1-3 and 11-13) and a general factor (Total score) loading on 12 items; the distress item (item 10) is not included (Flessner et al 2009; Flessner al., 2011; Bipeta et al 2013).*

*Measurement invariance*

Measurement invariance (MI) is present when a specific instrument (e.g., FAS-PR) measures the same construct across different groups (Cheung & Rensvold, 2002). If MI is established, then it can be confirmed that the participants across all groups interpret the individual items, as well as the underlying latent factors, in the same way. Conversely, failure to prove MI indicates that groups interpret the items differently and, as a consequence, factor means cannot be compared in a meaningful way (Jöreskog, 1971; Vandenberg & Lance, 2006).

Here we tested MI across raters (mothers vs fathers) to examine whether the results from the CFA were consistent across parents and allowed meaningful comparisons between them. MI was tested in MPlus version 8.4 using MLR estimator.

MI examines the change in the goodness-of-fit indices (GFI) when cross-group constraints are imposed on a measurement model in a hierarchical set of steps (Meredith & Teresi, 2006). Configural, metric, scalar, and measurement error invariance were tested. Configural invariance refers to whether the same CFA is valid in each group. Metric invariance—also called weak factorial invariance—concerns the equivalence of the factorial loadings across groups. Scalar (or strong factorial) invariance is assumed when the item intercepts and the factor loadings are equally constrained across groups. Finally, when testing residual (or strict factorial) invariance, also the variances of the residuals are constrained across groups.

We compared models following the recommendations of Chen (2007). That is, when sample size is adequate (total N > 300) and sample sizes are equal across the groups, like in our case (mothers n=209, fathers n=209, total N=418), the following criteria is suggested. For testing loading invariance, a change of ≤ -0.010 in CFI, supplemented by a change of ≥ 0.015 in RMSEA or a change of ≥ 0.030 in SRMR would indicate noninvariance; for testing intercept or residual invariance, a change of ≤ -0.010 in CFI, supplemented by a change of ≥ 0.015 in RMSEA or a change of ≥ 0.010 in SRMR would indicate noninvariance

*Post-hoc analyses multivariate linear regression*

Additional exploratory post-hoc analyses multivariate linear regressions were carried out to examine correlates of maternal and paternal FA using SDQ and DASS subscales instead of total scores for these measures. All significantly correlated variables were entered as independent variables and maternal and paternal FAS-PR scores as dependent variables.

**Results**

*Confirmatory factor analysis*

Table S1 presents the results of the CFA for the four different FAS-PR scoring models or factor structure used in the OCD literature. Models 1 and 2 were equivalent in terms of CFI, TLI and RMSEA. However, the lower AIC and BIC values in Model 4 suggested that a 12-item FAS-PR bi-factor factor structure, incorporating two subscales (*Avoidance of Triggers* and *Involvement in Compulsions*) and a *Total* FAS-PR score, fitted the data best, both for mothers and fathers. Therefore these 2 subscales, together with the *Total* FAS-PR score (12 items), were tested for measurement invariance.

| **Table S1. Confirmatory Factor Analyses results for the different Family Accommodation Scale- Parent Report (FAS-PR) models.** | | | | | | | | | |
| --- | --- | --- | --- | --- | --- | --- | --- | --- | --- |
| **Source** | **Model** | **χ^2^** | **df** | ***p*** | **CFI** | **TLI** | **RMSEA** | **AIC** | **BIC** |
| *Mother* | *1* | 66.451 | 54 | 0.119 | 0.990 | 0.986 | 0.033 | 8280.17 | 8447.29 |
|  | *2* | 241.644 | 65 | <.000 | 0.859 | 0.831 | 0.114 | 8462.10 | 8592.45 |
|  | *3* | 97.721 | 27 | <.000 | 0.906 | 0.874 | 0.112 | 5980.10 | 6070.34 |
|  | ***4*** | ***58.148*** | ***42*** | ***0.049*** | ***0.986*** | ***0.978*** | ***0.043*** | ***7713.91*** | ***7874.34*** |
| *Father* | *1* | 91.872 | 54 | 0.001 | 0.970 | 0.957 | 0.058 | 8082.67 | 8249.79 |
|  | *2* | 257.979 | 65 | <.001 | 0.848 | 0.818 | 0.119 | 8278.97 | 8409.32 |
|  | *3* | 106.840 | 27 | <.001 | 0.889 | 0.852 | 0.119 | 5838.64 | 5928.89 |
|  | **4** | **78.993** | **42** | **0.005** | **0.967** | **0.949** | **0.065** | **7559.92** | **7720.35** |
| *χ2, chi-square; df, degrees of freedom; p, p value; CFI, Comparative Fit Index; TFL, Tucker Lewis Index; RMSEA, Root Mean Square Error of Approximation; AIC, Akaike Information Criteria; BIC, Bayesian Information Criteria.*  *Best fitting model in bold.* | | | | | | | | | |

*Measurement invariance*

A configural invariance model was initially specified in which bi-factor models were estimated simultaneously within each group; factor means were fixed to 0 and factor variances were fixed to 1 for identification within each group. As shown in Table S2, the configural model had good fit, and thus a series of model constraints were then applied in successive models to examine potential decreases in fit resulting from measurement non-invariance.

Equality of the item factor loadings across groups was then examined in a metric invariance model in which the factor variances were fixed to 1 in mothers but were freely estimated in fathers; the factors means were fixed to 0 in both groups. All factor loadings were constrained to be equal across groups; all intercepts and residual variances were still permitted to vary across groups. The metric invariance model fit well (see Table S2) and did not result in a significant decrease in fit relative to the configural model based on changes in CFI, RMSEA and SRMR. The fact that metric invariance held indicates that the items were related to the latent factors equivalently across groups, or more simply, that the same latent factors were being measured in each group.

Equality of the unstandardized item intercepts across groups was then examined in a scalar invariance model. The factor means and variances were fixed to 0 and 1, respectively, for identification in mothers, but the factor means and variances were then estimated in the fathers. All factor loadings and item intercepts were constrained to be equal across groups; all residual variances were still permitted to differ across groups. The scalar invariance model did not hold since CFI was ≤-0.01 (i.e., -0.011) and SRMR was ≥0.01 (i.e. 0.01). Examination of the modification indices suggested a point of localized strain with the intercept of item 1; accordingly, a partial scalar invariance model was thus estimated in which the intercept for item 1 (“How often do you reassure your child?”) was allowed to differ between groups, resulting in a good-fitting model. The partial scalar invariance model did not fit significantly worse than the metric invariance model, indicating that partial scalar invariance did hold. The factor that partial scalar invariance (i.e., “strong invariance”) held indicates that both groups have the same expected item response at the same absolute level of family accommodation, or more simply, that the observed differences in item means between groups is due to factor mean differences only. The exception to this case is item 1, for which mothers were expected to have a higher item response than fathers at the same absolute level of family accommodation.

Equality of the residual variances across groups was then examined in a residual variance invariance model. As in the partial scalar invariance model, the factor mean and variance were fixed to 0 and 1, respectively, for identification in the mothers, but the factor means and variances were still estimated in the fathers. All factor loadings, item intercepts (except for item 1), and all residual variances (except for item 1) were constrained to be equal across groups. The residual variance invariance model fit well (see Table S2) and did not result in significant decrease in fit relative to the partial scalar invariance model. The fact that residual variance invariance held indicates that the amount of item variance not accounted for by the factor was the same across groups.

Since the threshold for non-invariance in the scalar model was just over the limit, and the model itself had an acceptable fit, we conducted the analyses including item 1. However, a sensitivity analysis was conducted excluding item 1.

| **Table S2. Fit indices of measurement invariance of bifactor model of FAS-PR across parents** | | | | | | | | | | |
| --- | --- | --- | --- | --- | --- | --- | --- | --- | --- | --- |
| **Model** | **χ^2^(df)** | **CFI** | **TLI** | **RMSEA** | **SRMR** | **Comp** | **ΔCFI** | **ΔRMSEA** | **ΔSRMR** | **Decision** |
| M1: Configural | 138.4 (84) | 0.976 | 0.962 | 0.056 | 0.035 | - | - | - | - | - |
| M2: Metric | 164.5 (105) | 0.974 | 0.967 | 0.052 | 0.048 | M1 | -0.002 | -0.004 | 0.013 | Accept |
| M3: Scalar | 197.8 (114) | 0.963 | 0.957 | 0.059 | 0.058 | M2 | -0.011 | 0.007 | 0.010 | Reject |
| M3a: Partial Scalar | 186.5 (113) | 0.968 | 0.962 | 0.056 | 0.051 | M2 | -0.006 | 0.004 | 0.003 | Accept |
| M4: Residual | 192.7 (124) | 0.970 | 0.968 | 0.051 | 0.053 | M3a | 0.002 | -0.005 | 0.002 | Accept |
| *Note. N = 418; group 1 mothers n = 209 (reference group); group 2 fathers n = 209.*  *Model M3a and M4, intercept of ítem 1 is allowed to be free between groups.* | | | | | | | | | | |

The only subscale **not** associated with FAS was SDQ Hyperactivity. All remaining subscales when entered in a model along with CY-BOCS, BDI, and CGAS.

| **Table S3. Pearson Correlation coefficients for candidate variables associated with FA** | | | | | | |
| --- | --- | --- | --- | --- | --- | --- |
| **Candidate variable** | **FAS-PR (mother) Total** | | | **FAS-PR (father) Total** | | |
|  | **n** | **r** | **p-value** | **n** | **r** | **p-value** |
| Age | 209 | -0.02 | 0.799 | 209 | -0.11 | 0.105 |
| Gender | 209 | 0.08 | 0.240 | 209 | 0.04 | 0.534 |
| Duration Illness | 192 | 0.08 | 0.257 | 192 | -0.01 | 0.921 |
| CY-BOCS total | 209 | 0.39 | <0.001 | 209 | 0.34 | <0.001 |
| CGAS | 179 | -0.32 | <0.001 | 179 | -0.34 | <0.001 |
| BDI | 192 | 0.29 | <0.001 | 192 | 0.23 | 0.001 |
| SDQ Total | 150 | 0.31 | <0.001 | 150 | 0.28 | <0.001 |
| SDQ Emotional | 150 | 0.23 | 0.004 | 150 | 0.19 | 0.022 |
| SDQ Hyperactivity | 150 | 0.11 | 0.179 | 150 | 0.09 | 0.291 |
| SDQ Conduct | 150 | 0.28 | 0.001 | 150 | 0.33 | <0.001 |
| SDQ Peer | 150 | 0.22 | 0.006 | 150 | 0.19 | 0.018 |
| SDQ Prosocial | 150 | -0.29 | <0.001 | 150 | -0.31 | <0.001 |
| DASS Total (mother) | 120 | 0.53 | <0.001 | 120 | 0.36 | <0.001 |
| DASS Anxiety (mother) | 120 | 0.44 | <0.001 | 120 | 0.29 | 0.001 |
| DASS Depression (mother) | 120 | 0.53 | <0.001 | 120 | 0.36 | <0.001 |
| DASS Stress (mother) | 119 | 0.49 | <0.001 | 119 | 0.36 | <0.001 |
| DASS Total (father) | 115 | 0.35 | <0.001 | 115 | 0.41 | <0.001 |
| DASS Anxiety (father) | 115 | 0.25 | 0.006 | 115 | 0.34 | <0.001 |
| DASS Depression (father) | 116 | 0.33 | <0.001 | 116 | 0.38 | <0.001 |
| DASS Stress (father) | 115 | 0.41 | <0.001 | 115 | 0.45 | <0.001 |
| *FAS-PR, Family Accommodation Scale Parent Report; CY-BOCS, Children Yale-Brown Obsessive-Compulsive Scale; CGAS, Children Global Assessment Scale; BDI, Beck Depression Inventory; SDQ, Strengths and Difficulties Questionnaire; DASS, Depression Anxiety Stress Scale.* | | | | | | |

*Post-hoc analyses multivariate linear regression*

The only subscale *not* associated with FAS was SDQ Hyperactivity. All remaining SDQ and DASS subscales when entered in a model along with CY-BOCS, BDI, and CGAS. For maternal FAS, the model accounted for 42% of the variance; only CY-BOCS total score was significantly associated with FAS (β=0.26, p=0.001). For paternal FAS, the model accounted for 37% of the variance, with CY-BOCS total score (β=0.20, p=0.013), SDQ conduct problems (β=0.21, p=0.012) and CGAS (β=-0.17, p=0.036) being significantly associated with FAS.

| **Table S4. Summary of post-hoc regression models examining associations between candidate variables (including subscales) and FAS-PR** | | | | | | | | |
| --- | --- | --- | --- | --- | --- | --- | --- | --- |
|  | **Maternal FAS-PR** | | | | **Paternal FAS-PR** | | | |
|  | **β** | **SE** | **z** | ***p*-value** | **β** | **SE** | **z** | ***p*-value** |
| CYBOCS total | 0.26 | 0.08 | 3.30 | **0.001** | 0.20 | 0.08 | 2.50 | **0.013** |
| SDQ Emotional | 0.07 | 0.08 | 0.80 | 0.426 | 0.05 | 0.08 | 0.57 | 0.566 |
| SDQ Conduct | 0.09 | 0.08 | 1.11 | 0.267 | 0.21 | 0.08 | 2.51 | **0.012** |
| SDQ Peer | 0.03 | 0.08 | 0.39 | 0.697 | -0.01 | 0.08 | -0.08 | 0.939 |
| SDQ Prosocial | -0.02 | 0.09 | -0.18 | 0.857 | -0.01 | 0.09 | -0.06 | 0.949 |
| DASS Depression (mother) | 0.23 | 0.14 | 1.65 | 0.099 | -0.01 | 0.15 | -0.06 | 0.956 |
| DASS Anxiety (mother) | -0.10 | 0.15 | -0.66 | 0.511 | -0.06 | 0.16 | -0.39 | 0.698 |
| DASS Stress (mother) | 0.21 | 0.14 | 1.50 | 0.133 | 0.10 | 0.15 | 0.68 | 0.497 |
| DASS Depression (father) | 0.07 | 0.13 | 0.53 | 0.599 | 0.15 | 0.14 | 1.08 | 0.280 |
| DASS Anxiety (father) | -0.12 | 0.15 | -0.83 | 0.404 | -0.02 | 0.16 | -0.13 | 0.897 |
| DASS Stress (father) | 0.21 | 0.16 | 1.26 | 0.208 | 0.21 | 0.18 | 1.18 | 0.240 |
| BDI | 0.01 | 0.09 | 0.11 | 0.916 | 0.03 | 0.09 | 0.33 | 0.742 |
| CGAS | -0.04 | 0.08 | -0.50 | 0.616 | -0.17 | 0.08 | -2.10 | **0.036** |
|  | R^2^=0.42, *p*<0.001 | | | | R^2^=0.37, *p*<0.001 | | | |
| *FAS-PR, Family Accommodation Scale Parent Report; CY-BOCS, Children Yale-Brown Obsessive-Compulsive Scale; CGAS, Children Global Assessment Scale; BDI, Beck Depression Inventory; SDQ, Strengths and Difficulties Questionnaire; DASS, Depression Anxiety Stress Scale; SE, standard error.* | | | | | | | | |
